# Psychometric properties of the situational procrastination scale of medical undergraduates: factor structure, reliability, and validity

**DOI:** 10.3389/fpsyt.2024.1440424

**Published:** 2024-10-25

**Authors:** Chunxiao Wang, You You, Nigela Ahemaitijiang, Zhuo Rachel Han

**Affiliations:** ^1^ Institute of Education, Tsinghua University, Beijing, China; ^2^ Institute of Medical Education, Peking University, Beijing, China; ^3^ Center for Counseling and Psychological Development, Tsinghua University, Beijing, China; ^4^ Beijing Key Laboratory of Applied Experimental Psychology, National Demonstration Center for Experimental Psychology Education, Faculty of Psychology, Beijing Normal University, Beijing, China

**Keywords:** college students, situational procrastination scale, academic procrastination, daily life procrastination, academic achievement

## Abstract

**Introduction:**

Procrastination is very common among college students, but there is a lack of consistency in the relationship between procrastination and academic achievement, which might be partly caused by the limitations of previous procrastination scales. The current study constructed the Situational Procrastination Scale (SPS) with two subscales, the Academic Situational Procrastination Scale (ASPS) and the Daily Life Situational Procrastination Scale (DSPS), by adapting previous procrastination scales.

**Method:**

The valid sample for data analysis included 2,094 medical undergraduates. After testing item discrimination, we conducted exploratory factor analysis, confirmatory factor analysis, and measurement invariance to examine the factor structures. Reliability (i.e., internal and test-retest reliability) and validity (i.e., concurrent, convergent, and discriminant validity) of the SPS were verified subsequently.

**Results:**

The ASPS included near lateness, lateness, procrastination on academic tasks before deadlines, and procrastination on academic tasks beyond deadlines, and measurement invariance across gender, household registration, and family financial status was found. The DSPS included procrastination on going out, consumption, routines, and communication, and had measurement invariance across grade, household registration, and family financial status. The results demonstrated adequate internal consistency, test-retest reliability, concurrent validity, convergent validity, and discriminant validity. Situational procrastination positively correlated with trait procrastination at a moderate or low level and negatively correlated with self-efficacy. Only procrastination on academic tasks before and beyond deadlines negatively predicted academic achievement.

**Discussion:**

The SPS could measure procrastination accurately and clarify the nexus between procrastination and academic achievement, which has implications for improving the academic warning system.

## 1 Introduction

Procrastination is a prevalent phenomenon in higher education. Estimates indicate that approximately 50% of college students nearly always procrastinate in academic areas and most of them desire to reduce their dilatory behaviors (1, 2). Procrastination is defined as delaying the start and/or completion of tasks (3–5) and a variety of studies proved that procrastination was associated with deleterious consequences. Specifically, procrastination could harm individual mental health, e.g., leading to negative emotions (6, 7), and has negative impacts on students’ learning, e.g., decreasing study satisfaction (8).

However, some researchers argued that procrastination could lead to desirable outcomes. Ferrari (9) suggested that some procrastinators obtained a pleasurable sensation through dilatory behaviors. To elucidate the positive effects of procrastination on individuals, Chu and Choi (10) proposed active procrastination, which was defined as the intention to procrastinate while still managing to complete tasks at the last minute. For example, active procrastination was positively linked to resource management strategy of college students (11). The key distinction between active and procrastination traditionally associated with deleterious consequences mentioned above is the fact that active procrastinators intentionally procrastinate yet still have the ability to complete tasks with satisfactory qualities before deadlines (12–14).

A variety of studies investigated the associations between academic achievement and procrastination among university students but the results were contentious. Procrastination had a negative relationship with academic achievement in some studies (15–24). However, other studies reported that procrastination did not correlate with academic achievement (1, 25–28). A positive link was even observed between procrastination and academic achievement (22, 29).

Measures mixed different types of procrastination in previous studies partially led to conflicting results, as the type of procrastination moderated the relationship between academic achievement and procrastination (13, 30). Specifically, using scales that fail to differentiate situational from trait procrastination could cause inconsistent relationships with academic achievement (17, 26) for situational procrastination was more likely to decrease academic achievement than trait procrastination (6). The conflation of academic and daily life situations in procrastination scales led to inaccurate associations between procrastination and academic achievement (31), because academic tasks are generally considered more critical to learning than life tasks (2, 32). Moreover, procrastination scales that mix meeting and missing deadlines might cause inconclusive correlations since the ability to meet deadlines had positive effects on academic achievement (12, 21, 22).

Existing procrastination scales with unsatisfactory psychometric properties could decrease the accuracy of measuring procrastination, which could also cause mixed results. For example, the widely used academic procrastination scale, Procrastination Assessment Scale-Students (PASS), mixed some non-academic tasks such as administrative tasks (2, 32) and its factor structure was not examined (1). Moreover, Ferrari (33) found that PASS demonstrated unsatisfactory internal reliability and test-retest reliability. The prevalent daily life procrastination scales, e.g., the General Procrastination Scale (GPS) and the Adult Inventory of Procrastination (AIP), were both viewed as unidimensional when they were developed and presented acceptable reliability (3, 9, 27, 33). However, factor analyses did not support their single-factor structures (34–36). Procrastination measured by the scales with unsatisfactory qualities had inconsistent relationships with academic achievement (15, 25, 31).

Given that existing procrastination scales have controversial contents (e.g., the conflation of situational versus trait procrastination, academic versus daily life situations, and meeting versus missing deadlines) and unsatisfactory psychometric properties, the current study aimed to construct a new situational procrastination scale by adapting the prevalent scales to overcome aforementioned limitations. Because students’ situational procrastination typically includes two situations, i.e., academic and daily life, and these two domains are considered relatively independent (32), the new scale is designed to contain two subscales that specifically target academic procrastination and daily life procrastination. Considering the potentially catastrophic consequences of procrastination among medical students, who are poised to assume significant responsibilities in their future professional careers (24), it is necessary to further investigate procrastination within this population. Employing medical undergraduates as the sample in the current study might enhance the discriminant validity of the newly constructed scale since the cohort procrastinated less than students from other majors (37). The large sample size (*N* = 2094) in this study could increase the robustness of the new scale’s quality. Therefore, the present study theoretically contributes to a deeper understanding of the various types of procrastination, enriches knowledge regarding the effects of procrastination on individuals, and addresses the deficiencies in existing scales. Practically, the new scale with satisfactory psychometric properties will measure procrastination minutely, clarify the effects of procrastination on the academic achievement, and identify the types of procrastination that are harmful to learning, which is useful to strengthen the quality supervision and diagnosis of medical students’ learning process.

## 2 Literature review

### 2.1 Measurement of procrastination

Based on the degree of situational specificity, procrastination contains situational procrastination and trait procrastination (38). Situational procrastination refers to deferring particular tasks and students’ situational procrastination could be further classified into academic procrastination and life procrastination (32). Trait procrastination, also called general or chronic procrastination, means postponing almost every task regardless of the context (6). Accordingly, there are three kinds of procrastination scales, e.g., academic, daily life, and trait procrastination scales.

The Procrastination Assessment Scale-Students (PASS) is a widely used academic procrastination scale. It estimates the procrastination in six tasks (i.e., writing papers, preparing for exams, reading assignments, administrative tasks, attending meetings, and general academic tasks) that rated on a 5-point Likert scale (1). However, some tasks (e.g., administrative tasks) of PASS do not belong to the academic area (2, 32), which might partially cause unsatisfactory reliability (33). To address this deficiency, Milgram et al. (32) chose three major academic tasks from PASS and developed Academic Procrastination Scale-Student Form (APS-SF). However, the sample used to develop it was small (*N* = 52) and there was no factor analysis to examine its factor structure. Other academic procrastination scales, e.g., the Aitken Procrastination Inventory (API) (39), contained items of trait procrastination (e.g., “*I often don’t finish tasks on time*”) and yielded unstable factor structures (40).

Daily life procrastination scales include the General Procrastination Scale (GPS) and Adult Inventory of Procrastination (AIP), both of which were designed as unidimensional scales (3, 9, 27, 33). GPS originally contained 20 items scored as true and false when it was developed (27), and it was later revised to a 15-item version scored on a 5-point Likert scale (41). Although it is named General Procrastination Scale, it contains various items targeting procrastination on daily life tasks (e.g., “miss concerts or sporting events”, “get up”, “return phone calls”, “arrive at the airport or station”). Therefore, some researchers, such as Ferrari and Johnson (40), utilized it to assess daily life procrastination. AIP consisted of 15 items scored on a 5-point Likert scale and it also mixed trait procrastination with life procrastination, similar to the GPS. These two scales did not have stable factor models (34–36).

Trait procrastination scales, such as the Tuckman Procrastination Scale (TPS), the Irrational Procrastination Scale (IPS), Pure Procrastination Scale (PPS), Unintentional Procrastination Scale (UPS), and the short form of GPS (GPS-9), are not involving specific situations of procrastination (42–45). Additionally, the New Active Procrastination Scale (NAPS) measures active procrastination across the general situation (12) and did not have stable factor structure (14).

In summary, the existing instruments have contributed to our understanding of procrastination but have some limitations. The reliability of PASS was unsatisfactory (33). Some scales (e.g., APS-SF, GPS, AIP, TPS, PPS, NAPS) had no clear factor structure examined by factor analyses with adequate sample size (14, 32, 34–36, 39, 42, 46, 47). The contents of some scales (e.g., PASS, API, GPS, AIP) are controversial (2, 9, 32, 38, 43).

### 2.2 Explanations of inconsistent relationships between procrastination and academic achievement

The contradictory links between students’ procrastination and academic achievement in previous studies might be due to different procrastination measures, academic achievement indices, data reporting methods (i.e., self-reported, and externally-gathered), and samples characteristics (30). The types of procrastination measured by different scales are crucial for understanding the relationship between procrastination and academic achievement, because after controlling for other moderators (i.e., academic achievement indices, data reporting methods, and sample characteristics), the link between procrastination and academic achievement remained inconclusive. For instance, some research that adopted multiple measures of procrastination simultaneously yielded inconsistent correlations with the same academic achievement index within the same sample (21, 22, 31). Previous meta-analyses analyzed the factors affecting the relationship between procrastination and academic achievement (13, 30). However, Kim and Seo (30) overlooked the differences in types of procrastination. Kooren, Van Nooijen (13) took a further step by categorically discussing the association of academic achievement with active versus traditional procrastination, yet still failed to distinguish situational versus trait procrastination, and academic versus life procrastination. We focused on procrastination types to analyze inconsistent relationships, which would help adapt the existing procrastination scales into a better version.

First, mixing different situations of procrastination could make the relationship inaccurate between procrastination and academic achievement. The relationship was inconsistent between university students’ academic achievement and procrastination assessed by PASS (15, 25, 31), API (17, 18, 26), and TPS (20, 21, 25, 28). These findings might be because some tasks of PASS made no reference to the academic area (2, 27, 32). TPS and some items of API targeted trait procrastination without distinguishing academic from other situations (32, 39, 42). The negative association of academic achievement with procrastination was significant when the procrastination scales focused on academic tasks (16, 31, 48, 49). Therefore, categorizing situations of procrastination would be useful to identify the effects on students’ academic achievement.

Second, ignoring “not meeting deadlines” of procrastination could interfere with the association between procrastination and academic achievement. Procrastination beyond deadlines seems to be more harmful to learning since the ability to meet deadlines was positively correlated with academic achievement (12, 21, 22). PPS and AIP once yielded a factor about “beyond deadlines”, which might lead to a negative relationship between procrastination measured by these two scales and academic achievement (19, 22). Thus, differentiating procrastination beyond and before deadlines would help recognize the exact association of procrastination with academic achievement.

The current study attempted to adapt existing procrastination scales into the new Situational Procrastination Scale (SPS) and the large sample size (*N* = 2094) could guarantee the robustness of the results. The SPS, with acceptable psychometric properties, will be able to distinguish the academic from daily life situations of undergraduates and classify the procrastination of whether or not to meet deadlines, thereby addressing limitations of existing scales (e.g., unsatisfactory reliability and validity, small sample size, and the conflation of various types of procrastination). Furthermore, the SPS will assess procrastination minutely and identify the link between specific procrastination and academic achievement accurately, which will help uncover types of procrastination affecting academic achievement more severely and contribute to knowledge of impacts of procrastination. This will serve as an early warning indicator of university students’ academic quality and further facilitate the intervention of harmful types of procrastination.

## 3 Method

### 3.1 Procedures

First, we generated the items of the SPS based on previous procrastination scales. Two rounds of pilot studies were conducted to ensure that all the items were clearly understood. Second, stratified sampling based on grade years was adopted in the main study to recruit participants from two public universities in mainland China. We adopted several methods to control the quality of the questionnaire before data analysis and the data cleaning process was described in Participants section. Item discrimination was tested to further guarantee the quality of each item. Third, we performed exploratory factor analysis (EFA) to explore factor structures and used confirmatory factor analysis (CFA) to ensure the factor models. After factor analyses, the measurement invariance across different groups was examined. Last, we verified the reliability (i.e., internal consistency and test-retest reliability) and validity (i.e., concurrent, convergent, and discriminant validity) of the SPS.

### 3.2 Participants

The questionnaire was distributed online through the Wenjuanxing platform from two universities in mainland China. Each sampled university appointed an online survey coordinator collecting students who volunteered to fill out the questionnaires. We provided the coordinators with requirements for the data collection, which included that the sample size at least five times the number of scale items. We collected 3,679 questionnaires in total and removed invalid ones prior to data analysis. Specifically, we deleted 1,487 questionnaires with patterns of answering (e.g., consecutive identical options), and eliminated 98 questionnaires with obvious errors in information (e.g., admission years of participants did not match their reported grades). We ultimately obtained 2,094 valid questionnaires which accounted for approximately 60% of the total questionnaires collected from the two universities.

Among 2094 participants, 1,443 (68.9%) came from University A, and 651 (31.1%) were from University B. 1,334 (63.7%) students were females, and 760 (36.3%) were males. Most of the participants were of Han ethnicity (98%), and 41 (2%) were ethnic minorities. The sample comprised 377 (18.0%) freshmen, 397 (19.0%) sophomores, 388 (18.5%) juniors, 430 (20.5%) fourth-year students, and 502 (24.0%) fifth-year students. 1,182 (56.4%) participants’ household registrations were rural, and 912 (43.6%) were from urban areas. 602 (28.7%) students reported living in families with financial difficulties, and 1,492 (71.3%) lived in families without financial difficulties.

We divided the sample (*N* = 2,094) into two independent halves. Subsample 1 (*N*1 = 1,031) was used to perform EFA while subsample 2 (*N*2 = 1,063) was used for CFA and measurement invariance. A total of 2,094 participants provided data for internal consistency and validity analyses. 180 students among the total sample took part in the retest 1.5 months later and provided data for assessing the test-retest reliability.

### 3.3 Measures

Three scales were adopted along with demographic information of the participants. The ranking of self-report grade point average (GPA) was considered an indicator of academic achievement, similar to a range of previous studies (20, 28, 50, 51). Students were asked to choose a percentage from 1% to 100% of their undergraduate GPA ranking in their grade year. The value 1% refers to the top position of GPA, and 100% means the bottom position of GPA. The smaller percentage of GPA ranking demonstrated better academic achievement. Given that procrastination was significantly connected with general self-efficacy (42, 52, 53), we added General Self-efficacy Scale (GSES) to verify the concurrent validity of the SPS. The GPS-9, a trait procrastination scale (45), was adopted to examine the convergent validity of the SPS.

#### 3.3.1 The situational procrastination scale

The SPS consists of two subscales, namely, the Academic Situational Procrastination Scale (ASPS), and the Daily Life Situational Procrastination Scale (DSPS). We originally designed 16 items of the ASPS and 14 items of the DSPS based on PASS, GPS, and AIP. The final adapted version included 12-item ASPS with four factors (i.e., near lateness, lateness, procrastination on academic tasks before deadlines, and procrastination on academic tasks beyond deadlines) and 13-item DSPS with four factors (i.e., procrastination on going out, consumption, routines, and communication). Each item of SPS was rated on a 5-point Likert scale ranging from 1 (*never*) to 5 (*always*) and items of the DSPS are reverse coded. Each factor is calculated by the average score of corresponding items to express the level of academic and daily life procrastination. The SPS demonstrated good reliability (McDonald’s omega = .760–.939) and validity.

#### 3.3.2 The short version of the general procrastination scale

The GPS-9 assessing trait procrastination has 9 items rated on a 5-point Likert scale ranging from 1 (*never*) to 5 (*always*). The unidimensional scale demonstrated good reliability (45). McDonald’s omega (ω) was.744 in the current study.

#### 3.3.3 General self-efficacy scale

The GSES is a 10-item unidimensional inventory measuring self-confidence in the ability to deal with a large variety of stressors. Items were rated on a 4-point Likert scale ranging from 1 (*not at all true*) to 4 (*exactly true*), with acceptable reliability (54). In the present study, omega was.925.

### 3.4 Data analysis

The software used in the study included SPSS 26, R 4.3.0, and Jamovi 2.3.18. Data was saved with SPSS 26, and when analyzing the data with R 4.3.0, it was first read using haven package in R 4.3.0. After testing item discrimination using Pearson’s correlation coefficients and critical ratio (CR) values with Jamovi 2.3.18 (55), we divided the sample (*N* = 2,094) into two independent halves using the random sampling procedure in SPSS 26. One half was used for EFA using Jamovi 2.3.18 and the other half was used for CFA and measurement invariance to verify the factor structures by R 4.3.0 with the lavaan package (56). We used Jamovi 2.3.18 to assess internal consistency by McDonald’s omega because it was more accurate than Cronbach’s alpha (57). Test-retest reliability, concurrent validity, and convergent validity were demonstrated by correlation coefficients which were analyzed with Jamovi 2.3.18. To test discriminant validity, we compared the average variance extracted (AVE), calculated according to its formula using a calculator, with the square of the factor correlations calculated by Jamovi 2.3.18. Additionally, we utilized R 4.3.0 to perform Shapiro-Wilk test on the scales as well as their factors to examine the normality of constructs before subsequent statistical analyses. The output showed that none of the constructs were normally distributed (*p* <.05). Therefore, non-parametric tests (e.g., Spearman’s correlation coefficient) were employed for the subsequent statistical analyses involving these constructs.

## 4 Results

### 4.1 Item generation

The SPS included the Academic Situational Procrastination Scale (ASPS) and the Daily Life Situational Procrastination Scale (DSPS). We designed items for ASPS based on the academic tasks from PASS (1) and designed items for DSPS based on life tasks from GPS and AIP (3, 9, 27, 33). These three existing scales were selected since they have been widely used among undergraduates (6) and they contained settlements of procrastination in academic and daily life situations of undergraduates. The newly designed items were evaluated through two rounds of pilot studies to ensure their applicability.

The ASPS measures academic procrastination and its items can be seen in **Table 1**. According to the PASS, college students’ academic tasks are listed as follows: writing term papers, preparing for exams, doing reading assignments weekly, performing administrative tasks, attending meetings, and performing general academic tasks. Given that not all undergraduates have to write papers, we changed this task to “doing assignments after class”. “Performing administrative tasks” was deleted because it does not belong to academic areas (32, 58). “Attending meetings” is not an academic task (2, 32), so we specified the task “meeting teachers” and “attending group study” which were belonged to academic domains. “Performing general academic tasks” was eliminated because general tasks could be represented by specific tasks (59). We added “attending lectures” and “self-directed learning” considering that lectures are often held on campus and that university students have the time to self-direct their studies. Therefore, the ASPS includes seven kinds of academic tasks, namely, doing assignments after class, preparing for exams, reading, meeting teachers, attending group study, attending lectures, and self-directed learning. There are two items for each academic task based on whether it is completed at deadlines. One item refers to delaying a task but finishing it before the deadline. The other item is about postponing a task beyond the deadline.

**Table 1:** Factor loadings for academic situational procrastination scale from EFA and CFA.

Factor/item	EFA loading (*N1 = 1,031*)	CFA loading (*N2 = 1,063*)
Procrastination on academic tasks before deadlines		
Item 11. I finished preparing for the exam in haste until the imminent deadline	.935	.724
Item 12. I did not finish preparing for the exam on time	.651	N/A
Item 9. I finished my homework in haste until the upcoming deadline	.479	.778
Item 13. I finish my reading assignments in haste until the imminent deadline	.439	.823
Lateness		
Item 6. I was late attending the exam	.839	.766
Item 2. I was late meeting my teacher	.780	.788
Item 4. I was late for the group study	.381	.697
Procrastination on academic tasks beyond deadlines		
Item 14. I did not finish my reading assignments on time	.840	.804
Item 16. I did not finish my self-directed learning tasks on time	.684	.748
Item 10. I did not finish my homework on time	.486	.646
Near lateness		
Item 3. I hurried to attend the upcoming group study when it was about to begin	.831	N/A
Item 7. I hurried to attend the lecture when it was about to begin	.596	.622
Item 1. I hurried to meet my teacher until the upcoming appointment	.472	.595
Item 5. I hurried to reach the examination room when it was about to begin	.407	.695

EFA, exploratory factor analysis; CFA, confirmatory factor analysis. Item 15 (“*I finished my self-directed learning tasks in haste until the imminent deadline*”) and Item 8 (“*I was late for the lecture*”) were excluded after EFA. Item 3 (“*I hurried to attend the upcoming group study when it was about to begin*”) and Item 12 (“*I did not finish preparing for the exam on time*”) were excluded after CFA.

The DSPS assesses daily life procrastination and its items can be seen in **Table 2**. Based on the GPS and the AIP, university students’ daily life tasks could be classified into physiological needs for clothing, food, housing, and transportation and the social need for communication. The items of the DSPS were designed from these aspects. Given that individuals do not need rational thinking when performing daily life tasks, and these tasks are relatively simple, many daily life tasks do not have strict deadlines. Thus, it is unnecessary to distinguish between meeting and not meeting deadlines for daily life procrastination.

**Table 2:** Factor loadings for daily life situational procrastination scale from EFA and CFA.

Factor/item	EFA loading (*N1 = 1,031*)	CFA loading (*N2 = 1,063*)
Procrastination on going out		
Item 2. I prepare my luggage and other items in advance when going out	.933	.961
Item 1. I arrive at the station or the airport early when going to another place	.896	.888
Item 3. When I need to pick up my friends or relatives at the airport or station, I arrive in advance	.886	.915
Procrastination on routines		
Item 12. I prepare to go to bed immediately when it is time to sleep	.778	.704
Item 14. I wash up immediately when it is time to do this	.728	.832
Item 11. I get up immediately when it is time to get up	.706	.774
Item 13. I have meals on time	.612	.725
Procrastination on consumption		
Item 4. If the articles for daily use were damaged, I would repair or replace them in time	.940	.834
Item 5. When I lack articles for daily use, I buy them on time	.770	.894
Item 6. I clean my room on time when it is dirty	.349	.670
Procrastination on communication		
Item 9. When I meet my friends, I arrive on time	.788	.852
Item 8. I handle missed phone calls or unread messages on time	.542	.703
Item 10. I would return the belongings in time if I borrowed them from others	.535	.688

EFA, exploratory factor analysis; CFA, confirmatory factor analysis. Item 7 (“*I do the laundry when my clothes are dirty*”) was excluded after EFA.

The designed items of ASPS and DSPS were reviewed and confirmed by researchers in the fields of psychology and education. We conducted the first pilot study to modify the items according to feedback from participants. To further verify whether the items were appropriate to medical students, the second pilot study was conducted among medical undergraduates. Following these procedures, the items were generated.

### 4.2 Item analysis

Pearson’s correlation coefficients and critical ratio (CR) values were presented in **Table 3** to indicate item discrimination of the SPS. Pearson’s correlation coefficients between each item of the ASPS and its total score ranged from.460 to.764, predicting good item discrimination. The items of the DSPS also demonstrated good discrimination (*r* = .538–.768). The CR values for items of ASPS and DSPS were analyzed respectively. We computed the cumulative scores for the ASPS items and ranked the total scores in descending order. The top 27% of participants by total score are classified as the high-scoring group, with corresponding scores 30–73. The bottom 27% of participants are classified as the low-scoring group, with corresponding scores ranging from 16 to 20. The CR values were determined by an independent sample t-test, comparing the scores for each item of ASPS between the high-scoring and the low-scoring groups. The CR values for ASPS ranged from 9.18 to 45.2 and all values were significant, demonstrating good item discrimination. For DSPS, the CR values were calculated using the same steps, with the high-scoring group scoring as 34–70 and the low-scoring group scoring as 14–23. As shown in **Table 3**, all the CR values for DSPS raged from 24.5 to 39.0 and were significant, predicting good item discrimination.

**Table 3:** Item discrimination of ASPS and DSPS (*N* = 2,094).

Item of ASPS	*r*	CR	Item of DSPS	*r*	CR
Item 1	.480^***^	15.0^***^	Item 1	.689^***^	30.4^***^
Item 2	.479^***^	10.3^***^	Item 2	.720^***^	31.3^***^
Item 3	.602^***^	22.3^***^	Item 3	.718^***^	31.5^***^
Item 4	.577^***^	16.6^***^	Item 4	.731^***^	39.0^***^
Item 5	.546^***^	16.0^***^	Item 5	.768^***^	37.3^***^
Item 6	.460^***^	9.18^***^	Item 6	.695^***^	36.9^***^
Item 7	.597^***^	21.2^***^	Item 7	.733^***^	36.0^***^
Item 8	.529^***^	14.8^***^	Item 8	.678^***^	33.3^***^
Item 9	.716^***^	41.1^***^	Item 9	.744^***^	34.1^***^
Item 10	.636^***^	23.1^***^	Item 10	.676^***^	24.5^***^
Item 11	.681^***^	42.8^***^	Item 11	.620^***^	29.4^***^
Item 12	.671^***^	38.2^***^	Item 12	.538^***^	25.0^***^
Item 13	.764^***^	45.2^***^	Item 13	.639^***^	29.0^***^
Item 14	.707^***^	32.5^***^	Item 14	.690^***^	34.7^***^
Item 15	.741^***^	40.9^***^			
Item 16	.650^***^	29.0^***^			

ASPS, Academic Situational Procrastination Scale; DSPS, Daily Life Situational Procrastination Scale; CR, critical ratio.

***p <.001.

### 4.3 EFA

To explore the factor structures of the ASPS and the DSPS, Subsample 1 was used for EFA using the extraction method of minimum residual in combination with an Oblimin rotation. Parallel analysis was adopted to determine the number of factors, and each factor had at least 3 items.

**Figure 1:**
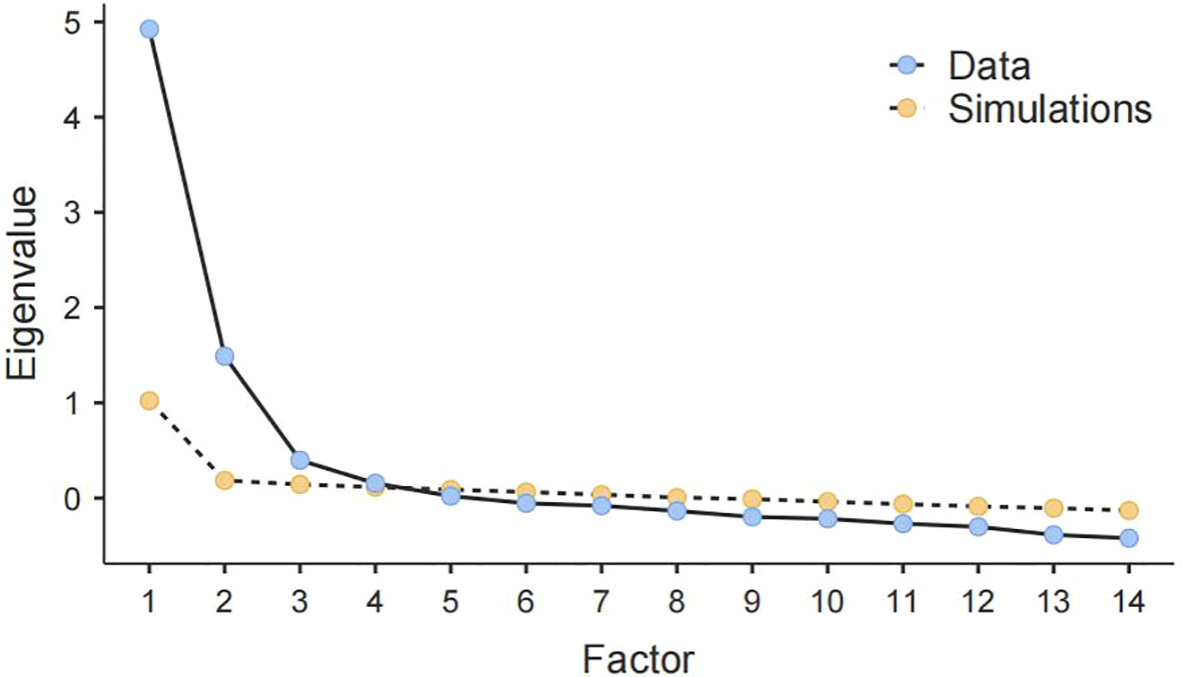
Scree plot of ASPS.

For the ASPS, correlation coefficients between items ranged from.120 to.693. Bartlett’s test of sphericity was significant and indicated correlation adequacy, χ^2^ = 6503, *p* <.001. The Kaiser-Meyer-Olkin measure of sampling adequacy was 0.881. Item 15 (“*I finished my self-directed learning tasks in haste until the imminent deadline*”) referred to delaying self-directed learning but still finishing it before the deadline in Chinese. However, it loaded on the factor of Procrastination on academic tasks beyond deadlines. Hence, Item 15 was deleted. Item 8 (“*I was late for the lecture*”) and Item 7 (“*I hurried to attend the lecture when it was about to begin*”) composed a factor with fewer than 3 items. Item 7 referred to nearly being late for the lecture and had a cross-loading higher than.4 under the Near lateness factor. After excluding Item 8, Item 7 only loaded on the Near lateness factor. Following the principles of parallel analysis (60), the first four factors of ASPS, which have eigenvalues larger than the corresponding random eigenvalues as shown in **Figure 1**, should be retained. Therefore, the four-factor model of the ASPS was generated, and every factor was named according to the main content reflected by the items. The variance explained by each factor ranged from 13.9% to 14.5%. The total variance explained by the four factors together reached 57.2%. All of the items retained had factor loadings higher than.38 as shown in **Table 1**. Although the factor loadings were typically greater than 0.4, Tabachnick and Fidell (2012, p. 654) suggested a minimum factor loading of 0.32 (61), and Hair et al. (2019, p. 151-152) suggested a cutoff of 0.3 (for the sample size larger than 350) (62). The lowest factor loading of ASPS was acceptable since it exceeded the minimum criteria and the sample size (*N1 =* 1,031) used for EFA was large enough, which could allow for more lenient criteria (62).

**Figure 2:**
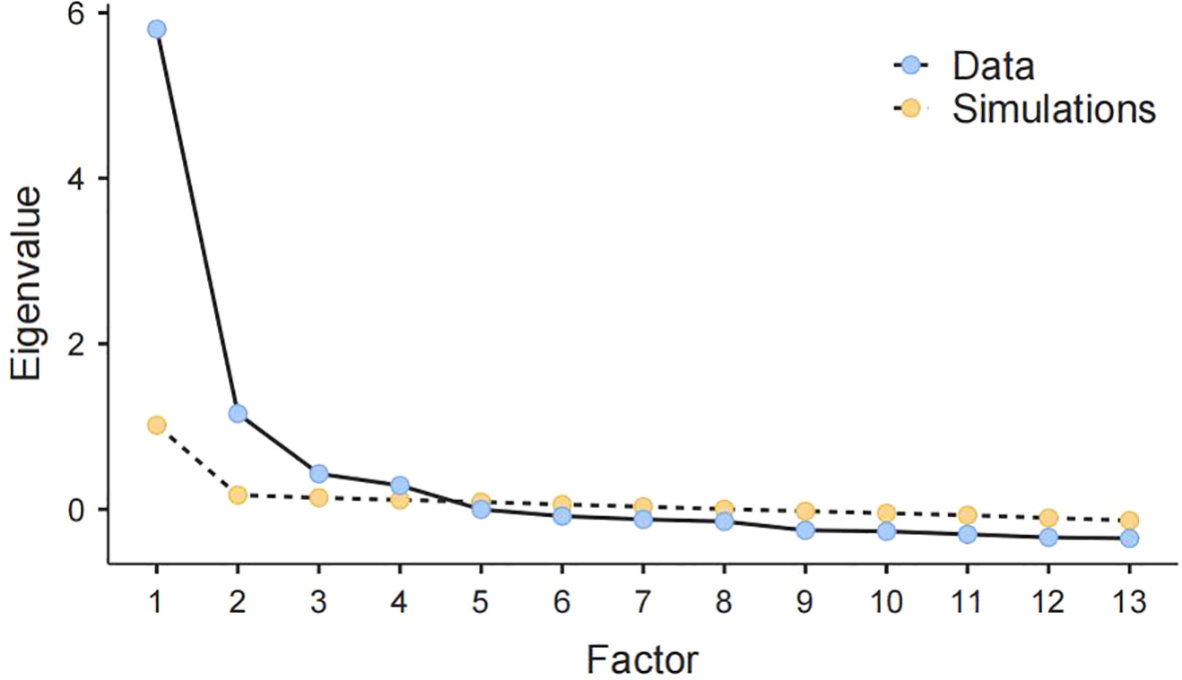
Scree plot of DSPS.

Concerning the DSPS, bivariate correlations between items ranged from.175 to.835. Bartlett’s test was significant (*p* <.001), and χ^2^ was 8397. The Kaiser-Meyer-Olkin test was 0.902. Item 7 (“*I do the laundry when my clothes are dirty*”) and Item 6 (“*I clean my room on time when it is dirty*”) composed a factor. Considering that doing the laundry and cleaning one’s room were similar and belonged to manual labor, we could keep one of them. Item 7 was eliminated because doing the laundry was not involved in previous daily life procrastination scales, i.e., GPS and AIP. According to the principles of parallel analysis (60), the eigenvalues of the first four factors for DSPS exceeded eigenvalues from simulations, as shown in **Figure 2**, supporting the four-factor structure. Thus, EFA of the DSPS yielded four factors. We named them based on the items’ contents, i.e., Procrastination on going out, on routines, on consumption, and on communication. The variance explained by each factor ranged from 13.7% to 20.2%. The cumulative variance explained by the four factors was 66.3%. **Table 2** shows factor loadings (.349–.940) for items of the DSPS from EFA. These loadings were acceptable as they were greater than the minimum factor loading criteria, and the sample size (*N1 =* 1,031) used for EFA was sufficiently large which could allow for more lenient criteria (61, 62).

### 4.4 CFA

Subsample 2 was used for CFA, employing the maximum likelihood-robust (MLR), to validate the alternative models which were shown in **Table 4**. We first integrated two kinds of situational procrastination (i.e., academic and daily life procrastination) as a single model and conducted CFA on its unidimensional structure, two-dimensional structure (i.e., including the two dimensions of academic and daily life procrastination), and higher-order structure. In the higher-order model, each of the two dimensions of academic and daily life procrastination contains four sub-dimensions yielded from EFA). Subsequently, academic procrastination and daily life procrastination were analyzed specifically. CFA was conducted on their single-factor and four-factor models respectively. We compared alternative structural models based on the acceptable criteria for model fit indices which included RMSEA less than.10 (63), CFI and TLI greater than.90, and SRMR less than.08 (56).

**Table 4:** Summary of goodness of fit for contrasting alternative models of situational procrastination scale (*N2 =* 1,063).

	Model	χ^2^	*df*	RMSEA	CFI	TLI	SRMR
Situational procrastination	One-factor Model	6679***	324	.160	.435	.358	.140
	Bi-factor Model	4915***	323	.135	.596	.561	.105
	Higher-order Model	1518***	315	.068	.902	.890	.073
Academic procrastination	One-factor Model	1715***	77	.178	.604	.532	.131
	Four-factor Model	520***	71	.095	.897	.867	.055
	Revised Four-factor Model	288***	48	.085	.928	.900	.053
Daily life procrastination	One-factor Model	2364***	65	.224	.597	.516	.126
	Four-factor Model	227***	59	.060	.974	.965	.041

RMSEA, root mean square error of approximation; CFI, comparative fit index; TLI, Tucker–Lewis index; SRMR, standardized root mean square residual.

***p <.001.

As observed in **Table 4**, for the integrated model of situational procrastination, the fit statistics of the higher-order model were best compared with those of the one-factor and bi-factor models. However, the fit indices of the higher-order model did not fully comply with the standards because TLI was lower than the acceptable standard of.90 (56).

The results of CFA conducted separately on academic procrastination and daily life procrastination were also presented in **Table 4**. For academic procrastination, although the four-factor model of the ASPS did not fit the data ideally with CFI and TLI less than.90 (56), this model still had better fit statistics than one-factor model. According to the modification indices, we found that Item 3 (“*I hurried to attend the upcoming group study when it was about to begin*”) was highly correlated with Item 2, 4, and 6, indicating that Item 3 had ambiguous meanings according to the participants’ understanding. Therefore, we removed Item 3. Item 12 (“*I did not finish preparing for the exam on time*”) of the ASPS conveyed different meanings from the other items of the factor Procrastination on academic tasks before deadlines. Thus, we eliminated Item 12. Consequently, the revised four-factor model fit the data well, with RMSEA value less than.10, CFI and TLI higher than.90, and SRMR value less than.08 (56). For daily life procrastination, the one-factor model did not meet the acceptable criteria with RMSEA higher than.10, CFI and TLI lower than.90, and SRMR higher than.08. In contrast, the four-factor model of DSPS fit the data very well, as suggested by RMSEA value less than.10, CFI and TLI values higher than.90, and SRMR value less than.08 (56).

Therefore, the revised four-factor model of ASPS and the four-factor model of DSPS were selected because their fit indices were the best among alternative models and met the acceptable criteria. The factor loadings from CFA on ASPS ranged from.595 to.823 (see **Table 1**) and the loadings of items for the DSPS from CFA were higher than.670 (see **Table 2**).

A multiple-group CFA was conducted to examine the measurement invariance across different groups. Three forms of measurement invariance were tested: configural (i.e., identical factor structure), metric (equal factor loadings across groups), and scalar (equal factor loadings and intercepts across groups). As shown in **Table 5**, for ASPS, chi-square difference tests between the configural, metric, and scalar models across household registration and family financial status were all nonsignificant, demonstrating strong measurement invariance. Chi-square difference tests across gender supported partial measurement invariance with comparison between configural and metric models nonsignificant. Regarding DSPS, chi-square difference tests across family financial status showed nonsignificant results, suggesting strong measurement invariance. Chi-square difference tests across grade and household registration supported partial measurement invariance since the comparisons between configural and metric models were nonsignificant. However, chi-square difference tests across gender were significant, which did not support the measurement invariance of DSPS across gender.

**Table 5:** Model fit for multiple group models and measurement invariance comparisons (*N2 =* 1,063).

	Model	χ^2^	Δχ^2^	*df*	RMSEA [90% CI]	CFI
Academic procrastination	Gender					
	Configural	348***	–	96	.084 [.075,.094]	.927
	Metric	337***	8.39	104	.081 [.071,.091]	.927
	Scalar	362***	24.8**	112	.080 [.071,.089]	.923
	Household registration					
	Configural	348***	–	96	.084 [.074,.093]	.930
	Metric	352***	15.2	104	.083 [.073,.092]	.926
	Scalar	368***	9.51	112	.080 [.071,.089]	.926
	Family financial status					
	Configural	363***	–	96	.087 [.077,.096]	.924
	Metric	341***	3.59	104	.082 [.072,.092]	.921
	Scalar	354***	4.36	112	.079 [.069,.088]	.927
Daily life procrastination	Gender					
	Configural	298***	–	118	.061 [.053,.070]	.972
	Metric	316***	17.8*	127	.061 [.052,.069]	.971
	Scalar	345***	31.0***	136	.061 [.053,.069]	.968
	Grade year					
	Configural	577***	–	295	.073 [.064,.082]	.962
	Metric	615***	41.8	331	.070 [.061,.078]	.961
	Scalar	675***	58.4*	367	.068 [.060,.076]	.958
	Household registration					
	Configural	303***	–	118	.062 [.053,.070]	.972
	Metric	314***	10.9	127	.060 [.052,.068]	.972
	Scalar	345***	34.5***	136	.061 [.053,.069]	.969
	Family financial status					
	Configural	283***	–	118	.059 [.050,.068]	.975
	Metric	288***	6.00	127	.056 [.047,.065]	.975
	Scalar	303***	13.6	136	.055 [.047,.063]	.975

RMSEA, root mean square error of approximation; CFI, comparative fit index.

Measurement invariance across grade years for ASPS was not presented because the covariance matrix of latent variables was not positive definite which might yield inaccurate results.

*p <.05. **p <.01. ***p <.001.

### 4.5 Reliability

The results illustrated satisfactory internal consistency and test-retest reliability for the SPS, as shown in **Table 6**. The acceptable criteria for internal consistency are greater than.60 for factors and.70 for scales. McDonald’s omega for the ASPS was.849, and omega coefficients for its factors were higher than.660. The internal consistency was good for the DSPS (ω = .908) and its factors (ω = .802–.939). The test-retest reliability correlation of the ASPS was.697, and the correlations for its factors ranged from.446 to.696. The test-retest reliability was adequate for the DSPS (*r* = .682) as well as its factors (*r* =.454–.662).

**Table 6:** Reliability of situational procrastination scale (*N* = 2,094, *N’* = 180).

Subscale/factor	McDonald’s ω	Test-retest reliability
**Academic Situational Procrastination Scale**	.849	.697
Procrastination on academic tasks before deadlines	.822	.696
Procrastination on academic tasks beyond deadlines	.772	.501
Lateness	.803	.446
Near lateness	.660	.491
**Daily Life Situational Procrastination Scale**	.908	.682
Procrastination on going out	.939	.454
Procrastination on consumption	.842	.662
Procrastination on routines	.838	.645
Procrastination on communication	.802	.520

*N’* was the sample for test-retest reliability. Test-retest reliability was demonstrated using Spearman’s correlation coefficients rather than Pearson’s correlation coefficients because constructs of Situational Procrastination Scale were not normally distributed. The bolded values indicate subscales, and the unbolded texts indicate factors of each subscale.

### 4.6 Validity

**Table 7** shows correlation coefficients among all variables measured in the study. Concurrent validity was tested by correlation coefficients among the factors of SPS, GSES, and GPA ranking. Academic procrastination and daily life procrastination had negative relationships with the general self-efficacy of medical students. The two factors (i.e., “*Procrastination on academic tasks before deadlines*” and “*Procrastination on academic tasks beyond deadlines*”) of ASPS were significantly related to general self-efficacy (PABF: *r_s_* = -.181, *p* <.001; PABY: *r_s_* = -.178, *p* <.001). However, the correlation coefficients of general self-efficacy with Lateness (*r_s_* = -.083) and Near lateness (*r_s_* = -.081) were relatively low.

**Table 7:** Spearman’s correlation coefficients among four factors of ASPS, four factors of DSPS, GPS-9, GSES, and GPA ranking (*N* = 2,094).

	PABF	PABY	Lateness	Near lateness	PG	PCON	PCOM	PR	GPS-9	GSES	GPA ranking
PABF	—										
PABY	.566***	—									
Lateness	.245***	.392***	—								
Near lateness	.366***	.304***	.447***	—							
PG	.079***	.233***	.267***	.228***	—						
PCON	.270***	.327***	.250***	.247***	.511***	—					
PCOM	.203***	.323***	.315***	.269***	.532***	.592***	—				
PR	.327***	.301***	.211***	.247***	.305***	.516***	.472***	—			
GPS-9	.643***	.554***	.326***	.363***	.277***	.492***	.434***	.432***	—		
GSES	-.181***	-.178***	-.083***	-.081***	-.187***	-.294***	-.273***	-.343***	-.247***	—	
GPA ranking	.178***	.153***	.035	-.009	.015	.067**	.073***	.054*	.121***	-.131***	—
*Mean*	2.25	1.62	1.14	1.39	1.77	2.25	1.85	2.4	2.41	2.68	46.4%
*SD*	0.921	0.704	0.387	0.575	1.010	0.859	0.796	0.786	0.576	0.529	0.268

PABF, Procrastination on academic tasks before deadlines; PABY, Procrastination on academic tasks beyond deadlines; ASPS, Academic Situational Procrastination Scale; PG, Procrastination on going out; PCON, Procrastination on consumption; PCOM, Procrastination on communication; PR, Procrastination on routines; DSPS, Daily Life Situational Procrastination Scale; GPS-9, Short version of General Procrastination Scale; GSES, General Self-efficacy Scale. Spearman’s correlation coefficients were adopted instead of Pearson’s correlation coefficients because factors of ASPS and DSPS, GPS-9, and GSES were not normally distributed.

*p <.05, **p <.01, ***p <.001.

**Figure 3:**
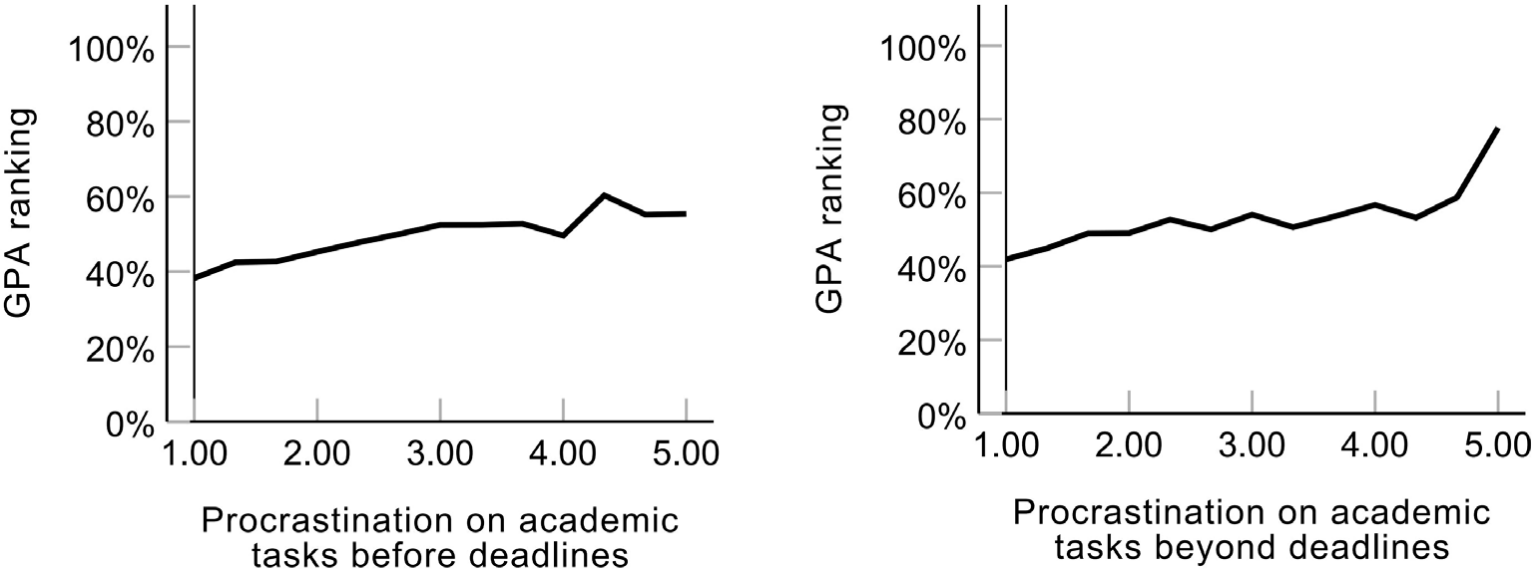
Prediction of procrastination on academic tasks on GPA ranking.

The positive correlation coefficients of ASPS factors with GPA ranking predicted negative relationships between academic procrastination and GPA because the value of the GPA ranking was greater, demonstrating the GPA was lower in the current study. Procrastination on academic tasks before deadlines (*r_s_* = .178, *p* <.001) and beyond deadlines (*r_s_* =.153, *p* <.001) had positive relationships with GPA ranking. **Figure 3** also shows the variation tendency of the GPA ranking as the frequency of the two factors (i.e., Procrastination on academic tasks before and beyond deadlines) changed, illustrating the negative effects of these two factors on academic achievement. Lateness, Near lateness, and factors of daily life procrastination had rather low correlation coefficients with GPA ranking (*r_s_* = -.009–.073).

Convergent validity was evaluated by correlation coefficients of SPS with GPS-9. Factors of ASPS and DSPS were positively associated with the GPS-9 score (*r_s_* =.277–.643, all with *p* <.001), as shown in **Table 7**. Most situational procrastination factors had a moderate correlation with GPS-9. Procrastination on academic tasks before and beyond deadlines had strong positive correlation coefficients (*r_s_* =.554–.643).

Discriminant validity was examined by comparing AVEs with squares of factor correlation coefficients. For the ASPS, *r_s_* ^2^ between Procrastination on academic tasks before deadlines and beyond deadlines was lower than AVEs of these two factors (*r_s_* ^2^ =.320; AVEs = .534–.603). The other squares of factor correlations were also lower than the corresponding factors’ AVEs (See **Table 8**). Concerning the DSPS, all squares of factor correlation coefficients were lower than the corresponding factors’ AVEs (*r_s_* ^2^ =.093–.350; AVEs = .564–.837), as shown in **Table 9**.

**Table 8:** Square of factor correlations and AVE of factors for ASPS (*N* = 2,094).

	*r_s_* ^2^				AVE
	PABF	PABY	Lateness	Near lateness	
PABF	—				.603
PABY	.320	—			.534
Lateness	.060	.154	—		.579
Near lateness	.134	.092	.200	—	.407

PABF, Procrastination on academic tasks before deadlines; PABY, Procrastination on academic tasks beyond deadlines; *r_s_^2^*, Square of Spearman’s correlation coefficient between factors; AVE, Average variance extracted from confirmatory factor analysis. Spearman’s correlation coefficients were adopted instead of Pearson’s correlation coefficients because factors of ASPS were not normally distributed.

**Table 9:** Square of factor correlations and AVE of factors for DSPS (*N* = 2,094).

	*r_s_* ^2^				AVE
	PG	PCON	PCOM	PR	
PG	—				.837
PCON	.261	—			.650
PCOM	.283	.350	—		.576
PR	.093	.266	.223	—	.564

PG, Procrastination on going out; PCON, Procrastination on consumption; PCOM, Procrastination on communication; PR, Procrastination on routines; *r_s_^2^*, Square of Spearman’s correlation coefficient between factors; AVE, Average variance extracted from confirmatory factor analysis. Spearman’s correlation coefficients were adopted instead of Pearson’s correlation coefficients because factors of DSPS were not normally distributed.

## 5 Discussion

The current study established a new SPS, including the ASPS with 12 items and the DSPS with 13 items. The items of SPS were generated based on existing scales (i.e., PASS, GPS, and AIP). After item analysis, factor models were confirmed by EFA and CFA, and measurement invariance was examined. Reliability (internal consistency and test-retest reliability) and validity (concurrent validity, convergent validity, and discriminant validity) were examined in sequence. The results illustrated the reasonable psychometric properties of the SPS, which are appropriate for medical undergraduates.

Item analysis was first conducted. The results demonstrated that all items of the SPS had adequate discrimination. The clear factor structures of the ASPS and the DSPS were then confirmed by EFA and CFA with an adequate sample size. Based on factor analyses, academic procrastination of college students contained four factors (i.e., Procrastination on academic tasks before deadlines, Procrastination on academic tasks beyond deadlines, Lateness, and Near lateness), which distinguished meeting and not meeting deadlines. Daily life procrastination of college students consisted of Procrastination on going out, Procrastination on consumption, Procrastination on routines, and Procrastination on communication. This made up for the gap that GPS and AIP did not have clear factor structures (34–36). Furthermore, strong measurement invariance for ASPS was supported across household registration and family financial status, and partial measurement invariance was supported across gender. These findings encourage the equivalent use of the ASPS for different groups. Regarding DSPS, strong measurement invariance was supported across family financial status and partial measurement invariance was found across grade and household registration. The results encourage the equal use of DSPS for families with different financial statuses, grades, and household registrations. However, chi-square difference tests did not support the measurement invariance of DSPS across gender, demonstrating that it is inadequate for comparing DSPS scores between males and females.

The reliability of SPS was found to be good. McDonald’s omegas for the subscales and their factors were higher than.660. The acceptable test-retest reliability of the subscales and each factor predicted adequate stability across time of the SPS. This was a contribution because the reliability of the PASS was not satisfactory (33).

Concurrent validity was satisfactory based on the association of SPS with general self-efficacy and GPA ranking. Previous research found that procrastination among university students had a negative relationship with self-efficacy (42, 52, 53), which was similar to the findings of the current study. This is understandable because individuals with higher self-efficacy would have more confidence in performing tasks, which could lead to less procrastination.

The correlation coefficients between GPA ranking and procrastination in attendance tasks (i.e., Lateness and Near lateness) and daily life procrastination were low in the present study. Procrastination on academic tasks within and beyond deadlines were all significantly associated with GPA ranking. That is, university students with better academic achievement procrastinate less on academic tasks. These results were similar to previous research findings. Only procrastination on academic tasks negatively correlated with the academic performance of university students (16, 31, 49), while non-academic procrastination and mixed types of procrastination had unstable correlations with academic achievement (1, 31). Furthermore, in the current study, procrastination on academic tasks was split into two factors. One factor (i.e., Procrastination on academic tasks before deadlines) means that people delay the start of academic tasks but still complete the academic tasks before the deadline, whereas the other (i.e., Procrastination on academic tasks beyond deadlines) means that procrastinators are not able to finish the academic tasks on time. These two factors were negatively associated with academic performance, which demonstrated that both delaying the start and the end of academic tasks would hurt the academic achievement of university students. Procrastination on academic tasks beyond deadlines is considered a more serious type of procrastination because the ability to meet deadlines had positive correlations with academic achievement (12, 21, 22). The GPA ranking changed more obviously for participants with high-frequency (frequency ≥ 4) procrastination on academic tasks beyond deadlines than for those with high-frequency procrastination on academic tasks before deadlines (See **Figure 3**). The different gradients of GPA ranking after the frequency ≥ 4 demonstrated that Procrastination on academic tasks beyond deadlines affected academic achievement more seriously than Procrastination on academic tasks before deadlines.

The convergent validity of SPS was good since the correlation coefficients between GPS-9 and SPS were similar to those in previous research. Magalhaes, Pereira (23) found that the correlation coefficient between the GPS-9 score and academic procrastination was.714. The correlation coefficient between TPS and PASS was.62 (25). Their correlation coefficients reached a moderate level rather than a high level since trait procrastination is different from situational procrastination (6). However, the correlation coefficient between trait procrastination and GPS score was high at.78 (44), which was partly because the GPS contains items involving trait procrastination.

The SPS showed good discriminant validity. Each square of the factor correlation coefficient was smaller than the corresponding AVEs of the factor pairs. The satisfactory discriminant validity of the ASPS proved that there was an obvious distinction between procrastination before deadlines and beyond deadlines. This result corresponded to previous research in which the AIP and PPS yielded a factor of lateness/not meeting deadlines (34, 35). The clear discriminant validity could also reflect that postponing academic tasks (e.g., doing assignments after class, preparing for exams, reading, self-directed learning) was different from postponing attendance tasks (e.g., meeting teachers, attending lectures), although these attendance tasks took place in the academic context. This result was consistent with the opinion of Milgram, Mey-Tal (32) and Rahimi and Hall (2). It was understandable why the total PASS score had inconsistent correlations with college students’ academic achievement (15, 31), but the negative relationship was significant between GPA and the PASS score limited to academic tasks (31). The clear discriminant validity of the DSPS demonstrated that there were differences among procrastination on various daily life tasks. This result could partly explain why studies did not support single-factor solutions of GPS and AIP (34–36).

Despite the contribution of the newly established SPS, several limitations of this study and future expectations need to be acknowledged. First, although we recruited participants from universities in mainland China, our research perspective was international as we came up with the research gaps from international references and samples. The SPS was constructed based on existing scales (i.e., PASS, GPS, AIP) which were used worldwide (15, 35, 36). According to studies using samples from other countries (e.g., Israel, Canada, the United States, the United Kingdom), some tasks (e.g., administrative tasks) of PASS do not belong to academic areas, instead, other tasks (e.g., exam preparation, assignments, reading) are the main academic tasks of undergraduates (2, 32, 53, 58, 59), which are similar to Chinese undergraduate academic context. Therefore, the SPS has the potential to assess procrastination of university students from Asia, North America, and Europe. However, we encourage future studies to further examine whether the SPS could be appropriate for students from other countries.

Second, all data were collected using self-report measures which were often used in previous studies because of convenience (20, 28). Students might overestimate their academic achievement due to the social desirability effect, which could decrease the coefficients between procrastination and academic achievement (30). Hence, the SPS might have more powerful effects on students’ learning than that in the current study. Moreover, as suggested in previous research (50), self-report GPAs were highly correlated with objective values. Similarly, this study also found that self-report GPA rankings had a close relationship (*r_s_* = -.596, *p* <.001) with graduate college entrance exam (GCEE) scores. The GCEE scores were collected when the students (*N* = 180) participated in the retest to provide data for assessing the test-retest reliability. 171 of them took part in the GCEE and reported their GCEE scores which were more objective than self-estimated GPA rankings. The close relationship between GPA rankings and GCEE scores suggested the GPA rankings were reliable to some extent.

Third, measurement invariance was not supported for DSPS across gender, suggesting that the measurement of DSPS might not be equivalent for males and females. As a result, comparisons of DSPS scores between males and females should be interpreted with caution. Future studies need to further examine and ensure the measurement invariance for DSPS across gender when comparing differences in daily life procrastination between males and females.

In summary, the SPS has two subscales, namely, the ASPS and the DSPS. Both have demonstrated satisfactory psychometric properties. The SPS will be useful for measuring college students’ procrastination in different situations, and the ASPS will further distinguish procrastination on academic tasks before and beyond deadlines. Because procrastination was significantly linked to negative outcomes in education, such as college dropout intentions (64), the SPS will be helpful to discover dysfunctional learning behaviors. Some studies aimed to intervene procrastination of students (65). The SPS could be adopted as a valid instrument to identify the harmful types of procrastination and assess the intervention effects. Therefore, we can use the SPS as an estimated tool to monitor college students’ learning and establish relevant intervention programs to overcome the harmful types of procrastination to promote academic quality.

## Data Availability

The raw data supporting the conclusions of this article will be made available by the authors, without undue reservation.
